# A Novel Non-Coding RNA CsiR Regulates the Ciprofloxacin Resistance in *Proteus vulgaris* by Interacting with *emrB* mRNA

**DOI:** 10.3390/ijms221910627

**Published:** 2021-09-30

**Authors:** Hongyang Zhang, Tongzhen Song, Chuhan Qin, Haijin Xu, Mingqiang Qiao

**Affiliations:** 1 College of Biotechnology and Food Science, Tianjin University of Commerce, Tianjin 300134, China; zhyang@tjcu.edu.cn; 2 The Key Laboratory of Molecular Microbiology and Technology, Ministry of Education, College of Life Sciences, Nankai University, Tianjin 300071, China; 2120201049@mail.nankai.edu.cn (T.S.); cq21@ic.ac.uk (C.Q.); 3 College of Life Sciences, Shanxi University, Taiyuan 030006, China

**Keywords:** non-coding RNA, CsiR, *emrB*, ciprofloxacin resistance, *Proteus vulgaris*

## Abstract

Bacterial non-coding RNAs (ncRNAs) play important regulatory roles in various physiological metabolic pathways. In this study, a novel ncRNA CsiR (ciprofloxacin stress-induced ncRNA) involved in the regulation of ciprofloxacin resistance in the foodborne multidrug-resistant *Proteus vulgaris* (*P. vulgaris*) strain P3M was identified. The survival rate of the CsiR-deficient strain was higher than that of the wild-type strain P3M under the ciprofloxacin treatment condition, indicating that CsiR played a negative regulatory role, and its target gene *emrB* was identified through further target prediction, quantitative real-time PCR (qRT-PCR), and microscale thermophoresis (MST). Further studies showed that the interaction between CsiR and *emrB* mRNA affected the stability of the latter at the post-transcriptional level to a large degree, and ultimately affected the ciprofloxacin resistance of P3M. Notably, the base-pairing sites between CsiR and *emrB* mRNAs were highly conserved in other sequenced *P. vulgaris* strains, suggesting that this regulatory mechanism may be ubiquitous in this species. To the best of our knowledge, this is the first identification of a novel ncRNA involved in the regulation of ciprofloxacin resistance in *P. vulgaris* species, which lays a solid foundation for comprehensively expounding the antibiotic resistance mechanism of *P. vulgaris*.

## 1. Introduction

Bacterial non-coding RNAs (ncRNAs) are a kind of RNA which cannot be translated into functional proteins but that have important regulatory functions [1,2,3]. A large number of studies have shown that ncRNAs are widely involved in the regulation of bacterial oxidative stress resistance [4,5], biofilm formation [6,7,8], osmotic pressure [9,10], virulence factor expression [11,12], bacterial antibiotic resistance [13,14,15], and many other physiological processes, among which the research of ncRNAs on the regulation of antibiotic resistance has become an important issue in recent years due to global drug resistance emergence. In view of their regulatory flexibility and low metabolic burden, ncRNAs have become one of the most versatile biobricks in the field of synthetic biology and metabolic engineering [16,17,18]. According to a number of studies, bacterial ncRNAs play regulatory roles in different modes, the most widely known of which are functioning via the interaction with the target mRNA [5,19,20].

Ciprofloxacin, part of the third generation of synthetic quinolone antibacterial drugs with broad spectrum antibacterial activity and good bactericidal effect, is widely used in the treatment of a variety of human and animal diseases caused by gram-negative bacteria [21,22]. With its heavy use, however, corresponding resistant bacteria gradually emerged and quickly spread and transferred in the natural environment, showing an alarming situation [23,24]. It is thereby imminently important to clarify the regulatory mechanism of ciprofloxacin resistance when developing new alternative drugs. Much research exists on ciprofloxacin resistance in bacteria, but few studies focused on the involvement of bacterial ncRNAs in this regulatory process, which was our focus in this study.

*Proteus vulgaris* (*P. vulgaris*), as one of the most common bacterial species of the genus *Proteus*, is an important opportunistic pathogen both environmentally and clinically, frequently causing gastrointestinal infection, urinary tract infection, and other diseases [25,26]. Inevitably, with the extensive use of clinical drugs, especially quinolones, for the treatment of related diseases, the appearance of resistant *P. vulgaris* has become a worsening problem, posing an important threat to human health [27,28,29,30]. In addition, as *P. vulgaris* widely exists in water, soil, spoilt food, animal intestinal tracts, and other common environmental media, possessing strong reproduction and transmission ability, its drug resistance is a very difficult issue to confront.

We previously identified a *P. vulgaris* strain, P3M, from the intestinal tract of *Penaeus vannamei* and clarified its multidrug resistance and evolutionary characteristics [30]. In this study, we further identified a novel ncRNA designated as CsiR from P3M and confirmed its regulatory role in the regulation of ciprofloxacin resistance through its interaction with *emrB* mRNA. This study reveals the important role of ncRNA in the drug resistance regulation process in *P. vulgaris* species, and provides theoretical basis for future clarification of the mechanism of multidrug resistance of genus *Proteus*.

## 2. Results

### 2.1. Response of ncRNA CsiR to Ciprofloxacin

**Figure 1:**
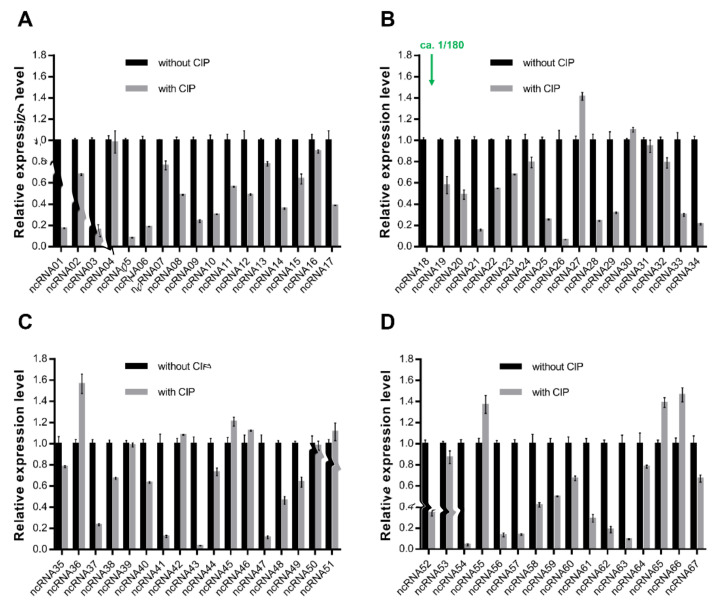
(**A**–**D**) Relative mRNA expression levels of 67 ncRNAs with the presence or absence of ciprofloxacin treatment. Expression data were obtained from three independent repeated tests (*n* = 3).

Preliminary studies showed there are 43 quinolone resistance genes and 67 ncRNAs in the P3M genome [30]. Since ncRNAs play important regulatory roles in the regulation of bacterial antibiotic resistance, it is of great importance to explore whether there are ncRNAs with regulatory functions in the P3M genome. The expression of these ncRNAs was detected under the treatment of 2 mg/L ciprofloxacin for 30 min (Figure 1). Most ncRNAs were downregulated to varying degrees compared to the expression of the untreated groups, among which ncRNA18 changed most significantly, showing a sharp decline with its relative expression level, at only 1/180 of the control (Figure 1B).

**Figure 2:**
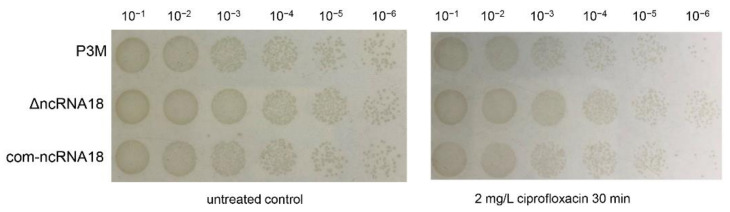
Spot growth assays of wild-type P3M, ncRNA18 deletion mutant (∆ncRNA18), and ncRNA18 complemented strain (com-ncRNA18), with or without ciprofloxacin treatment. Survival data were obtained from three independent repeated tests (*n* = 3).

In order to further verify the function of ncRNA18, we constructed the deletion mutant strain, complemented the strain of ncRNA18, and detected the survival rate of these two strains as well as the wild-type strain P3M under the ciprofloxacin treatment condition. The survival rate of the mutant strain ∆ncRNA18 largely improved compared to the wild-type strain, while the phenotype was retained to P3M in the complemented com-ncRNA18 strain (Figure 2). Hereby, we preliminarily concluded that ncRNA18 could respond to ciprofloxacin pressure signals, and its deletion mutant could improve the ciprofloxacin resistance of P3M to a certain level. Therefore, ncRNA18 was tentatively named as ciprofloxacin stress-induced ncRNA (CsiR) in this study.

### 2.2. The Involvement of emrB in the Regulation of Ciprofloxacin Resistance

**Figure 3:**
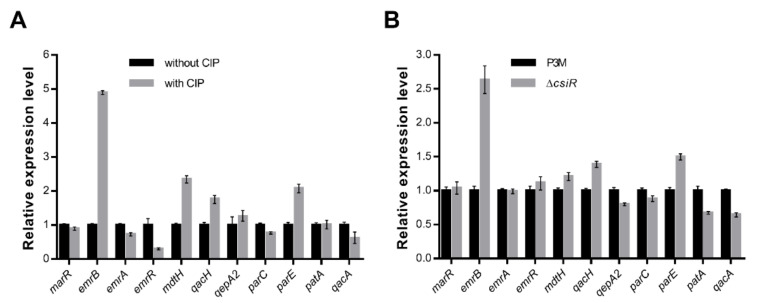
Relative mRNA expression levels of selected quinolone resistance genes with and without ciprofloxacin treatment (**A**), in wild-type and mutant backgrounds (**B**). Data were obtained from three independent repeated tests. Expression data were obtained from three independent repeated tests (*n* = 3).

ncRNA usually interacts with target mRNA to play specific regulatory roles [19,20]. In view of the importance of CsiR in improving ciprofloxacin resistance to P3M, we speculated there was a specific target gene interacting with CsiR in the P3M genome. Previous studies showed that there are 43 quinolone resistance genes in the P3M genome [30], of which 11 are nonpoint mutation resistance genes. We thus focused on these 11 genes and detected their expression. The expressions of *emrB*, *mdtH*, *qacH*, and *parE* were visibly upregulated under the 2 mg/L ciprofloxacin treatment compared with the untreated group, among which *emrB* expression showed the most significant change, with nearly five times as much as that of the control group, suggesting its potential as a target gene (Figure 3A). We then examined the expressions of the above genes in the ∆*csiR* mutant strain (Figure 3B). Similarly, under the same condition, *emrB* expression remained the most significant. It is noteworthy that the expression level of *emrB* in the mutant strain was upregulated compared with that of P3M, indicating that ncRNA CsiR regulates the expression of *emrB* in a negative way. Bioinformatics prediction results showed that among all the candidate targets that may bind with CsiR, only *emrB* belongs to the category of quinolone resistance genes, and the binding site is located at its 5′ UTR region. Accordingly, we think that *emrB* is highly likely the target gene of CsiR involved in the regulation of ciprofloxacin resistance of P3M, but further verification is needed.

**Figure 4:**
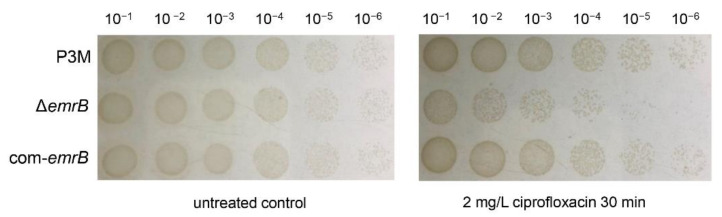
Spot growth assays of wild-type P3M, *emrB* deletion mutant strain (∆*emrB*), and *emrB* complemented strain (com-*emrB*), with or without the treatment of ciprofloxacin. Survival data were obtained from three independent repeated tests (*n* = 3).

EmrB, the important integral protein of cell membranes, has been well-studied and identified as a multidrug efflux pump [31,32]. To further clarify the function of *emrB* in P3M, we constructed the deletion mutant strain ∆*emrB* and complemented the com-*emrB* strain, and detected their survival phenotypes. As Figure 4 shows, the survival of ∆*emrB* significantly reduced compared to P3M under 2 mg/L ciprofloxacin treatment, while the phenotype of com-*emrB* basically restored to that of P3M, indicating that *emrB* is an important functional gene that can significantly promote the development of ciprofloxacin resistance of P3M. Two new complemented strains, ∆*csiR* + com-*emrB* and ∆*emrB* + com-*csiR,* were constructed to verify the interaction specificity between CsiR and *emrB*. The overexpression of *emrB* under the *csiR* deletion background further improved bacterial survival compared with the *csiR* mutant strain (Figure S1), while *csiR* overexpression in the *emrB* mutant strain did not lead to significant changes in the survival rate compared with ∆*emrB* (Figure S2). These results indicated that there is a specific interaction mechanism between CsiR and *emrB*, and CsiR alone could not regulate the ciprofloxacin resistance of P3M.

### 2.3. Interaction Mechanism between CsiR and emrB mRNA

The full length of CsiR is 77 bp, and its possible binding site with *emrB* mRNA is 66–74 bp (-AAUCUAGAA-) at its 3′ end according to the predicted result, as shown in the highlighted area in Figure S3. The full length of *emrB* coding region is 1518 bp, and the base-pairing site with CsiR is located at its 5′ UTR (-UUCUGGUUU-) (Figure S4).

**Figure 5:**
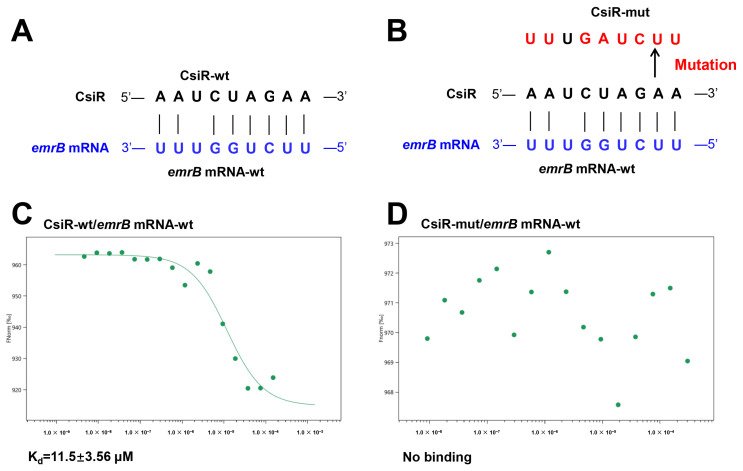
Binding affinity between CsiR and *emrB* mRNA. (**A**,**B**) Schematic diagram and binding or mutation of the base-pairing sites between CsiR and *emrB* mRNA, respectively. The binding sites of *emrB* mRNA and the mutated sites of CsiR are marked in blue and red, respectively. (**C**,**D**) Determination of the affinity of CsiR-wt and CsiR-mut binding to *emrB* mRNA, respectively. Affinity data were obtained from three independent repeated tests (*n* = 3).

To validate the possible interaction between CsiR and *emrB* mRNA, RNA oligonucleotide sequences of 30 nt wild-type CsiR (CsiR-wt), 30 nt site-mutated CsiR (CsiR-mut), and 30 nt wild-type *emrB* mRNA (*emrB* mRNA-wt) were synthesized in vitro (Figure 5A,B), and their binding affinity was verified by microscale thermophoresis (MST) experiments. CsiR-wt was capable of binding with *emrB* mRNA-wt, and an S-shaped curve was obtained by fitting, exhibiting a dissociation constant of 11.5 ± 3.56 μM (Figure 5C). However, there was no binding trend between *emrB* mRNA-wt and CsiR-mut mutating in the predicted base-pairing site of CsiR (Figure 5D). These results suggested that there is a specific interaction between CsiR and *emrB* mRNA at the predicted region.

**Figure 6:**
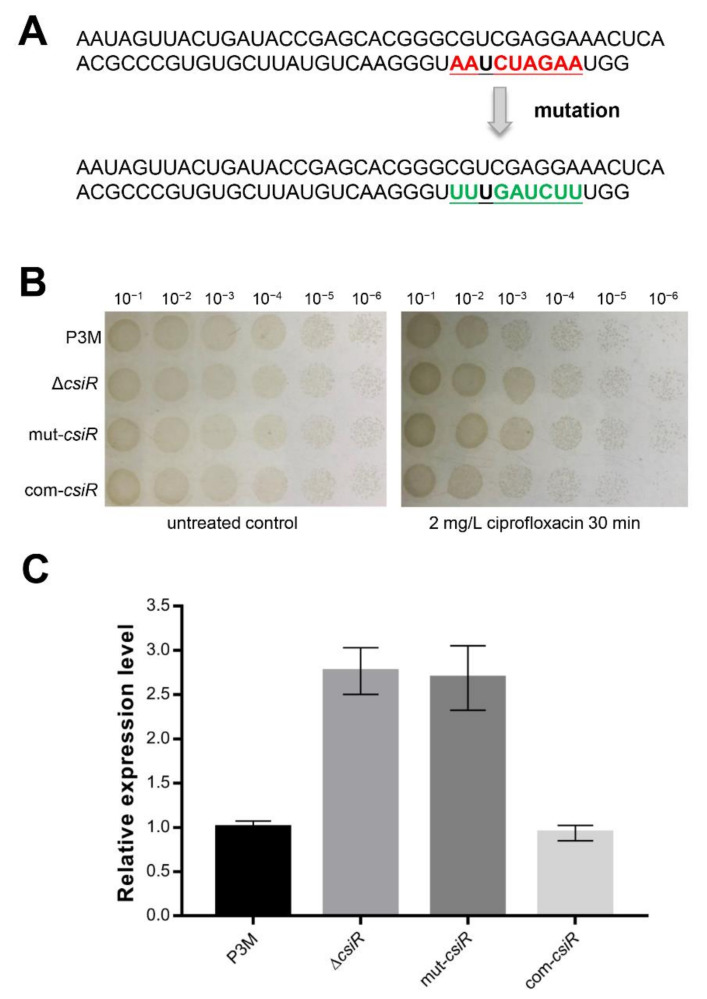
In vivo validation of the binding sites of CsiR. (**A**) Schematic diagram of the binding sites mutation of CsiR. (**B**) Spot growth assays of wild-type P3M, *csiR* deletion mutant strain (∆*csiR*), *csiR* complemented stain with mutations (mut-*csiR*), and *csiR* complemented strain (com-*emrB*) with or without the treatment of ciprofloxacin. (**C**) Relative mRNA expression levels of *emrB* in different backgrounds. Data were obtained from three independent repeated tests. Survival and expression data were obtained from three independent repeated tests (*n* = 3).

This interaction between CsiR and *emrB* mRNA needs to be further verified by in vivo experiments. The full-length CsiR carrying mutated binding sites was obtained by PCR (Figure 6A), which were then transferred into ∆*csiR* to construct the new complemented strain mut-*csiR*. As presented in Figure 6B, the survival rate of mut-*csiR* was almost the same as that of ∆*csiR*, and higher than that of the wild-type P3M and the complemented strain com-*csiR*. Correspondingly, the expression of *emrB* in the mut-*csiR* strain increased to the level of that in ∆*csiR* and was significantly higher than that in P3M and com-*csiR* (Figure 6C), indicating that this region is the binding site that interacts with *emrB* mRNA.

**Figure 7:**
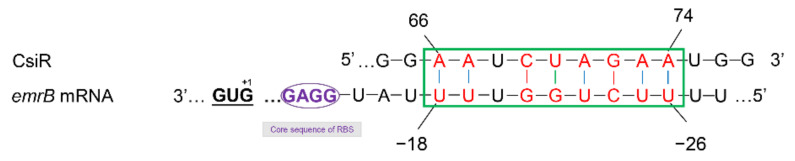
Base-pairing interaction between CsiR and *emrB* mRNA. The binding sites between CsiR and *emrB* mRNA are marked in red. The core sequence of RBS is marked in purple.

The binding sites of CsiR and *emrB* mRNA were further analyzed and explored to better understand the interaction mechanism between them. The binding site of CsiR is located at 66–74 bp of its 3′ end, while the binding site of *emrB* mRNA is located at 26–18 bp of its 5′ UTR region (Figure 7). Notably, sequence -GGAG- exists next to the binding site of *emrB* mRNA. -GGAG- is the core sequence of SD sequence within the ribosome binding sites (RBS) in prokaryotes, which can be accurately recognized by 16S rRNA and guide the initiation process of translation. Previous studies have shown that secondary stem-loop conformation is formed in the specific region of target mRNA once the mRNA sequence within the range of 55 bp near the SD sequence binds to ncRNA, which directly affects the recognition of the SD sequence by 16S rRNA, and thus affects the initiation of translation to some extent [33,34,35,36]. Therefore, we think that, in this study, CsiR also interacted with *emrB* mRNA in this way to contribute to the regulation of ciprofloxacin resistance in P3M.

**Figure 8:**
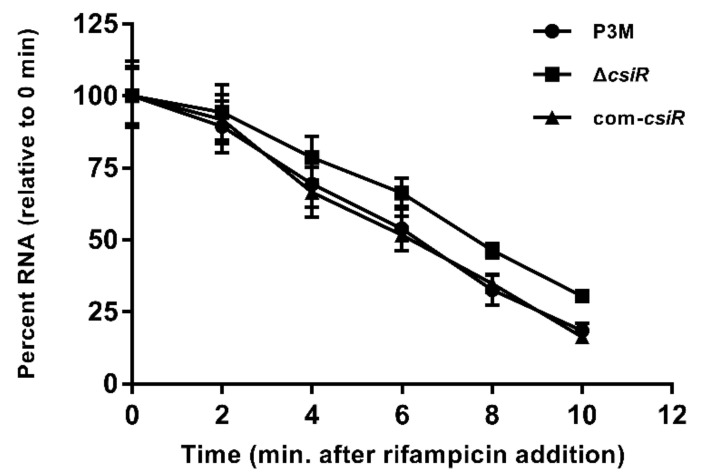
Determination of the half-life of *emrB* mRNA. Data were obtained from three independent repeated tests. Half-life data were obtained from three independent repeated tests (*n* = 3).

To verify the above conclusion, the stability of the *emrB* transcription was determined. Under 2 mg/L ciprofloxacin treatment, the half-life of *emrB* mRNA in ∆*csiR* was stable at about 8 min, significantly higher than that of the P3M and com-*csiR* strains with values of 6 min (Figure 8), indicating that the mutation of CsiR directly led to the high intracellular abundance of *emrB* mRNA. In other words, the interaction between CsiR and *emrB* mRNA significantly reduced the stability of the latter at the post-transcriptional level, further affecting the translation process.

### 2.4. Species Specificity of CsiR Regulatory Mechanism

**Figure 9:**
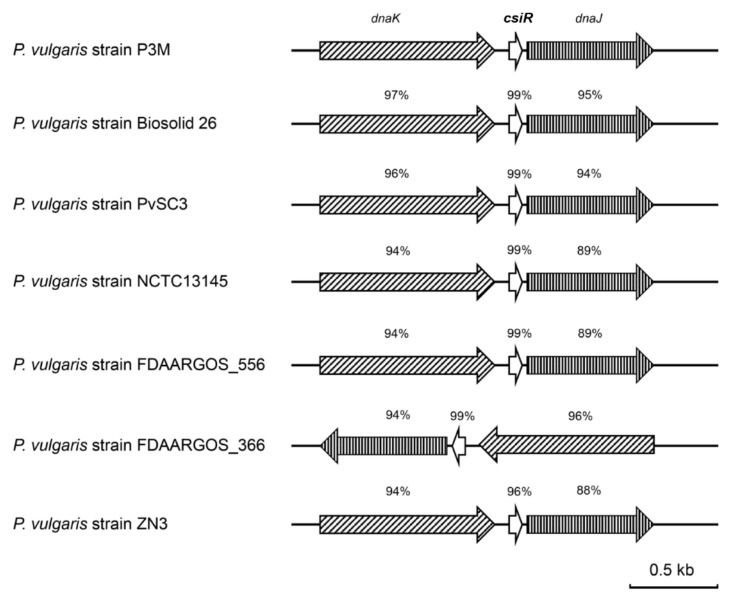
Sequence alignment analysis of *csiR* in all sequenced *P. vulgaris* strains.

**Figure 10:**
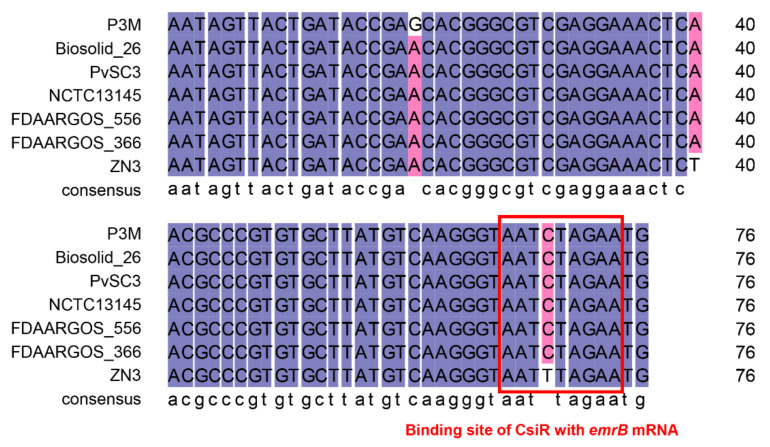
Sequence alignment of *csiR* in P3M with homologous sequences in all sequenced *P. vulgaris* strains. Sequences marked with red boxes are the binding sites of CsiR interacting with *emrB* mRNA.

**Figure 11:**
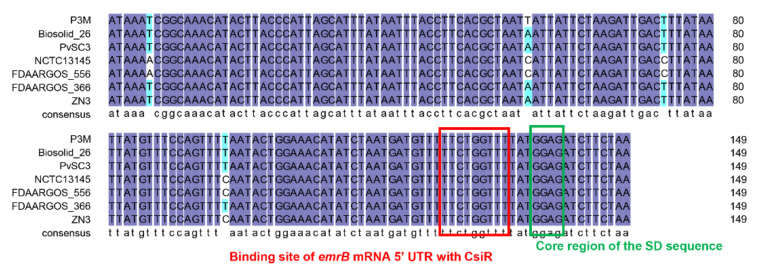
Sequence alignment of *emrB* mRNA 5′ UTR in P3M with homologous sequences in all sequenced *P. vulgaris* strains. Sequences marked with red and green boxes are the binding sites of *emrB* mRNA interacting with CsiR and the core region of the SD sequence, respectively.

Studies have shown that the regulation mechanism of ncRNA may be specific to bacterial species [5,37]. According to the result of a BLAST analysis, we found that *csiR* only exists in *P. vulgaris* species that have been sequenced, with a sequence consistency of up to 96% or more (Figure 9), implying that CsiR may be a *P. vulgaris* specific ncRNA. Subsequently, we analyzed the binding sites of CsiR interacting with *emrB* mRNA in all seven *P. vulgaris* strains, and the sequences marked by the red box in Figure 10 shows that this region is 100% conserved in almost all strains except for ZN3, with a substitution of C by T. In addition, sequence alignments of the binding site of *emrB* mRNA were also performed in all of the seven *P. vulgaris* strains. As shown by the sequence marked in the red box in Figure 11, the interaction region of *emrB* mRNA in P3M is highly conserved in other *P. vulgaris* strains, likewise with a sequence consistency of up to 100%. As expected, the SD core sequences -GGAG- in the same location were also equally consistent in the other six strains.

Since CsiR and *emrB* mRNA as well as their interaction sites are highly conserved in all sequenced *P. vulgaris* strains, we speculated that the regulatory mechanism of ciprofloxacin resistance in P3M may be pervasive with species-specific characteristics, which further research needs to verify.

## 3. Discussion

Studies have obtained strong evidence that antibiotics are the direct driving force behind bacterial resistance. As part of the third generation of synthetic quinolone antibacterial drugs, ciprofloxacin is widely used in clinical treatment and agricultural cultivation, inhibiting the normal function of bacterial DNA helicase and causing irreversible damage, leading to good antibacterial effects on *Pseudomonas aeruginosa*, *Escherichia coli*, *Proteus vulgaris*, and many other Enterobacteriaceae bacteria [38,39,40,41,42]. We previously isolated a multidrug-resistant *P. vulgaris* strain P3M from the intestinal tract of *Penaeus vannamei,* with 67 ncRNAs being identified [30]. Given the powerful role of ncRNAs in regulating antibiotic resistance [14,43], we emphasized the regulatory effect of ncRNA on ciprofloxacin resistance in common pathogenic bacterium P3M, which enabled CsiR to be identified and elucidated.

Typically, the binding of ncRNA to target mRNA can enhance the stability of the latter by unfolding the stem-loop conformation, exerting a positive regulatory function of ncRNA [5,19,37]. Different from the usual research cases, CsiR presented a negative regulatory function as its deficient strain showed increased ciprofloxacin resistance compared with wild-type P3M and complemented the strain com-*csiR* (Figure 2). Further bioinformatics analyses and target prediction results showed that *emrB* encoding an important multidrug efflux pump was the possible target gene of CsiR, which was confirmed by MST and point mutation experiments (Figure 5 and Figure 6). The binding sites of *emrB* mRNA are adjacent to the core sequence (-GGAG-) of RBS, which largely impedes the recognition and binding of RBS by 16S rRNA [34,35,36]. This conclusion was confirmed by the data presented in the half-life measurement experiment (Figure 8), namely in that the binding of CsiR to *emrB* mRNA significantly reduces the stability of the latter at the post-transcriptional level. We also found that the absence of CsiR affects the polymyxin B resistance and erythromycin resistance of P3M in a large part (data not shown), implying that CsiR is likely to be a multifunctional regulatory RNA, thus indicating its non-redundancy and its importance in resisting adverse abiotic stress in the external environment.

Another point that needs to be emphasized in this study is that as the sequence alignment results showed that the binding sites of CsiR and *emrB* mRNA share quite a high-sequence consistency among all sequenced *P. vulgaris* strains (Figure 10 and Figure 11), the mechanism of CsiR in regulating ciprofloxacin resistance may be *P. vulgaris* specific. Given the long process of evolution, bacteria, including *P. vulgaris,* inevitably encounter various kinds of survival pressures, especially antibiotics. As a result, bacteria have evolved complex and sophisticated regulatory mechanisms to better adapt to disadvantageous environments [44,45,46]. We suspected that this evolutionarily conserved trait, to a large extent, makes *P. vulgaris* more adaptable to modern medical and natural environments. Certainly, this speculation needs to be proved by subsequent studies.

In conclusion, we report for the first time a novel regulatory ncRNA CsiR involved in the regulation of ciprofloxacin resistance in the *P. vulgaris* strain P3M by binding with the 5′ UTR region of *emrB* mRNA and its evolutionary conservation, providing important theoretical guidance for further evolutionarily elucidating the important physiological functions of ncRNA, and clarifying the pervasive mechanism of antibiotic resistance regulation of the *P. vulgaris* species.

## 4. Materials and Methods

### 4.1. Strains and Culture Conditions

Strains and plasmids used in this study are listed in Table S1. All strains were cultured in Luria-Bertani (LB) medium (DINGGUO, Beijing, China) at 37 °C. Tetracycline (10 μg/mL) (DINGGUO, Beijing, China), ampicillin (100 μg/mL) (DINGGUO, Beijing, China), or streptomycin (50 μg/mL) (DINGGUO, Beijing, China) was added to the medium when needed.

### 4.2. Construction of csiR and emrB Deletion Mutants

In order to verify the functions of CsiR and *emrB*, the deletion mutant strains were constructed by homologous recombination in this study. The P3M genome was used as the template to amplify 500 bp sequences upstream and downstream of *csiR* or *emrB*. The two 500 bp sequences obtained in the previous step were fused by PCR to obtain the 1000 bp sequence, which was then ligated to the suicide plasmid pEX18Tc [47]. The recombinant plasmids were then transformed into *E. coli* DH5α competent cells, and correct plasmids were extracted and transferred into *E. coli* S17 [48]. After that, the correct *E. coli* S17 transformants were introduced into wild-type P3M by bi-parental mating. Correct mutant strains were screened by homologous recombination single and double crossovers, and were finally verified by sequencing.

### 4.3. Construction of csiR and emrB Complemented Strains

The *csiR* and *emrB* complemented strains were constructed based on the deletion mutant strains. Complete sequences of target genes, including both promoter and terminator regions, were amplified using the P3M genome as a template and ligated to clone plasmid pDN18 [49]. The recombinant plasmids were then transferred into *E. coli* DH5α competent cells and verified by colony PCR and sequencing. After, correct plasmids were extracted and transferred into *E. coli* S17. Correct *E. coli* S17 transformants were conjugated with the deletion mutant strain by bi-parental mating as well. Positive transformants were screened on LB agar plates with ampicillin (100 μg/mL) and tetracycline (10 μg/mL), and verified by colony PCR and sequencing.

### 4.4. Spot Growth Assays

The survival rate of P3M and its derivatives to ciprofloxacin was determined as previously described with minor modifications [5,19]. Strains were grown overnight in LB broth at 37 °C and were transferred into fresh LB broth and cultured to an OD600 of 0.6. The obtained bacterial solution was transferred into fresh LB broth again, with or without the treatment of 2 mg/L ciprofloxacin for 30 min. Gradient dilution was performed on the treated cultures, and 5 μL droplets were spotted onto the LB agar plate. The plates were then incubated at 37 °C for 24 h until colony growth was observed.

### 4.5. Quantitative Real-Time PCR

The relative mRNA expression levels of the selected genes in this study were determined by quantitative real-time PCR (qRT-PCR), using the StepOnePlus^TM^ real-time PCR system (ABI, Shrewsbury, MA, USA) as previously described [50]. In this study, total bacterial RNA was firstly extracted and reverse-transcribed into cDNA, which was diluted to 100 ng/μL. qRT-PCR reaction was then carried out in a reaction system consisting of 1 μL template cDNA (100 ng/μL), 10 μL 2× SYBR Green qPCR Master Mix (Vazyme, Nanjing, China), 1 μL upstream and 1 μL downstream primers (10 μM), and 7 μL ddH_2_O according to the manufacturer’s instructions. The gene-specific primers listed in Table S2 were designed based on the genome sequence of P3M and the 16S rRNA gene was used as the endogenous reference gene to normalize the expression of target genes in each cDNA template. The relative expression level was calculated by the comparative threshold cycle (2^-∆∆CT^) method, and each result was independently repeated three times.

### 4.6. ncRNA Target Prediction

The interaction between CsiR and its possible target mRNA was predicted by the sTarPicker prediction method [51] and the RNA Predator webserver [52].

### 4.7. RNA Secondary Structure Prediction

The secondary structures of CsiR and *emrB* mRNA in this study were predicted by the Mfold Web Sever according to instructions [53].

### 4.8. Microscale Thermophoresis Assays

The binding between CsiR and *emrB* mRNA was determined by microscale thermophoresis (MST), which is a technique used to analyze the interactions between biomolecules such as nucleic acids and proteins based on changes in the size, hydration layer, and electric charge of biomolecules due to the binding between molecules [37,54,55]. In this study, the 30 bp single-stranded RNA fragments CsiR-wt, CsiR-mut, and *emrB* mRNA-wt containing a binding region were synthesized in vitro*,* among which CsiR-wt and CsiR-mut were used as targets and labeled with 5′ FAM. *emrB* mRNA-wt was used as ligand with no label. A sample of 4 μL containing 500 nM target labeled with 5′ FAM (CsiR-wt/CsiR-mut) and increasing concentrations of non-labeled ligand (*emrB* mRNA-wt) were loaded on treated, standard capillaries. The measurements were then carried out using a Monolith NT.115 instrument (NanoTemper, Munich, Germany) at 26 °C in diethyl pyrocarbonate (DEPC)-treated water with 40% excitation power and medium MST-Power. The dissociation constants (K_d_) were calculated as previously described [37,54,55].

### 4.9. Half-Life Determination of emrB mRNA

Strains were grown overnight in LB broth and transferred into new LB broth. Ciprofloxacin with final concentration of 2 mg/L was added immediately into the cultures as the OD600 reached 0.6, which were fully mixed and incubated at 37 °C for 30 min. Rifampicin (40 mg/mL) (M057501) was added immediately after the ciprofloxacin treatment to stabilize the intracellular RNA content at a specific point in time. Samples of 1 mL were collected at different times (0, 2, 4, 6, 8, and 10 min). Then, 400 μL RNA (R0901) was added, fully mixed, and incubated for 5 min at room temperature to slow the RNA attenuation process by inhibiting the intracellular RNase activities. Samples were again centrifuged followed by the total RNA extraction. The half-life of *emrB* mRNA was detected by qRT-PCR as described above. Data are presented as percentages of mRNA levels relative to time point zero.

## Data Availability

Not applicable.

## Supplementary Materials

The following are available online at https://www.mdpi.com/article/10.3390/ijms221910627/s1.
