# Supplementary material for: A Novel Non-Coding RNA CsiR Regulates the Ciprofloxacin Resistance in Proteus vulgaris by Interacting with emrB mRNA
Source: Int J Mol Sci. 2021 Sep 30;22(19):10627. doi: 10.3390/ijms221910627 (PMC8508932; doi:10.3390/ijms221910627)
Supplement: Supplementary file 1 [file ijms-22-10627-s001.zip › Table S1.pdf]

Table S1. Strains and plasmids used in this study.

Strains or plasmids [a]	Characteristics	Sources
***P. vulgaris* strains**		
P3M	Wild-type strain	[30]
Δ*csiR* (ΔncRNA18)	CsiR (ncRNA18) deletion mutant strain	This study
com-*csiR* (com-ncRNA18)	CsiR (ncRNA18) complemented strain, Tc^r	This study
mut-*csiR*	CsiR (ncRNA18) complemented strain with mutations of binding site, Tc^r	This study
Δ*emrB*	*emrB* deletion mutant strain	This study
com-*emrB*	*emrB* complemented strain, Tc^r	This study
Δ*csiR* + com-*emrB*	*emrB* complemented strain in the *csiR* deficient background, Tc^r	This study
Δ*emrB* + com-*csiR*	*csiR* complemented strain in the *emrB* deficient background, Tc^r	This study
***E. cherichia* strains**		
Escherichia coli DH5α	Competent cell for cloning	CWBIO Company (CW0808S)
Escherichia coli S17	Mobilizing donor strain, Sm^r	[47]
**Plasmids**		
pEX18Tc	Suicide plasmid used for construct the deletion mutant strain, Tc^r	[48]
pDN18	Broad-spectrum clone plasmid used for the construction of functional complemented strain, Tc^r	[49]
