# Supplementary material for: A Novel Non-Coding RNA CsiR Regulates the Ciprofloxacin Resistance in Proteus vulgaris by Interacting with emrB mRNA
Source: Int J Mol Sci. 2021 Sep 30;22(19):10627. doi: 10.3390/ijms221910627 (PMC8508932; doi:10.3390/ijms221910627)
Supplement: Supplementary file 1 [file ijms-22-10627-s001.zip › Table S2.pdf]

Table S2. Primers used in this study.

Primer [a]	Sequence (5'-3') [b]	Purpose
18up-F	ac**GAATTC**TCAGCGAAAGATAAAAACAGTGGT	Amplification primers of 500 bp homologous sequence upstream of 5' end of ncRNA18 gene
18up-R	ttgCACAGATGAGTTACCGCGGC	
18down-F	gcggtaactcatctgtgCAAAAAGAGATTTTTACGAAGTACTCG	Amplification primers of 500 bp homologous sequence downstream of 3' end of ncRNA18 gene
18down-R	cc**AAGCTT**ATGACAAGTAGAACAGGTTTCTGCTG	
18-m1-F	GTCTCCCCATGCGAGAGTAG	Single crossover validation primers for homologous recombination of ncRNA18 mutant
18-m1-R	AGAGCTACACCGACGAGCTG	
18-m2-F	TCAGCGAAAGATAAAAACAGTGGT	Double crossover validation primers for homologous recombination of ncRNA18 mutant
18-m2-R	ATGACAAGTAGAACAGGTTTCTGCT	
18-com-F	ac**GAATTC**TCAGCGAAAGATAAAAACAGTGGT	Amplification and validation primers of complete ncRNA18 gene
18-com-R	cc**AAGCTT**ATGACAAGTAGAACAGGTTTCTGCTG	
18mut-up-F	ac**GAATTC**TCAGCGAAAGATAAAAACAGTGGT	Amplification primers of 500 bp homologous sequence with mutations upstream of 5' end of ncRNA18 gene
18mut-up-R	AAGATCAAAACCCTTGACATAAGCACACGGG	
18mut-down-F	TTTGATCTTTGGCAAAAAGAG	Amplification primers of 500 bp homologous sequence with mutations downstream of 3' end of ncRNA18 gene
18mut-down-R	cc**AAGCTT**ATGACAAGTAGAACAGGT	
emrB-up-F	ac**GAATTC**GCAATTATGCGTAGTTGTGCACA	Amplification primers of 500 bp homologous sequence upstream of 5' end of *emrB* gene
emrB-up-R	ggcgaggtgtaaagTGCTATTAACGTAGGCCCTATAAATAA	
emrB-down-F	atagcaCTTTACACCTCGCCTTTATTGTCC	Amplification primers of 500 bp homologous sequence downstream of 3' end of *emrB* gene
emrB-down-R	cc**AAGCTT**TTCAAGTCGGTGCACAAGTCTC	
pgsA-m1-F	GTCTCCCCATGCGAGAGTAG	Single crossover validation primers for homologous recombination of *emrB* mutant
pgsA -m1-R	AGAGCTACACCGACGAGCTG	
pgsA-m2-F	GCAATTATGCGTAGTTGTGCACA	Double crossover validation primers for homologous recombination of *emrB* mutant
pgsA -m2-R	TTCAAGTCGGTGCACAAGTCTC	
pgsA-com-F	ac**GAATTC**GCAATTATGCGTAGTTGTGCACA	Amplification and validation primers of complete *emrB* gene
pgsA-com-R	cc**AAGCTT**TTCAAGTCGGTGCACAAGTCTC	
Prov-16S-F	TCGCCTAGGTGAGCCTTTAC	Amplification primers of internal reference gene for the expression detection of functional gene in P3M
Prov-16S-R	CGAGCGGTAACAGGAGAAAG	
ncRNA01-F	TTGCATTCGCTTTACGCT	Amplification primers of ncRNA01 for qRT-PCR detection
ncRNA01-R	ACAGGACTTGGTAAAACGCA	
ncRNA02-F	CAGTAAACTGGTAGCTG	Amplification primers of ncRNA02 for qRT-PCR detection
ncRNA02-R	ATGCTACCGCCCCAGGCT	
ncRNA03-F	AAGGGAATCTGGTGCAAAGC	Amplification primers of ncRNA03 for qRT-PCR detection
ncRNA03-R	GCAGGTCTTCGGACTCTAGG	

ncRNA04-F	CAATCTGGAACAAGCTGAAAA	Amplification primers of ncRNA04 for qRT-PCR detection
ncRNA04-R	TTGACGTATCACAAATTCAAGG	
ncRNA05-F	GTTTGAGTATCCTGAAAACG	Amplification primers of ncRNA05 for qRT-PCR detection
ncRNA05-R	CTGCTATACGGGCCACTATG	
ncRNA06-F	CTTTAACAATCTGGAACAAGCTG	Amplification primers of ncRNA06 for qRT-PCR detection
ncRNA06-R	AGAGTACTTTTCAGATTTGAGATTTTG	
ncRNA07-F	CATTCCACTCCTTATGACAGCA	Amplification primers of ncRNA07 for qRT-PCR detection
ncRNA07-R	TCAGGTCTACAGACGGATGGT	
ncRNA08-F	CATTCCACTCCTTATGACAGCA	Amplification primers of ncRNA08 for qRT-PCR detection
ncRNA08-R	CTCAACGTTTCGTCCTGTTG	
ncRNA09-F	TCTCGAATGGCAAGTTAC	Amplification primers of ncRNA09 for qRT-PCR detection
ncRNA09-R	CAGTATTCTATTCTCTG	
ncRNA10-F	GGATAGCTGAATTAGAGATTGGC	Amplification primers of ncRNA10 for qRT-PCR detection
ncRNA10-R	GCATCACGACCAAATTCAGC	
ncRNA11-F	CCCTCGCGACTAATGAAAAT	Amplification primers of ncRNA11 for qRT-PCR detection
ncRNA11-R	CCTGTTGGCCCCTTTTCTA	
ncRNA12-F	CAATCTGGAACAAGCTGAAAAA	Amplification primers of ncRNA12 for qRT-PCR detection
ncRNA12-R	TTGATGTCAAAACACATTCAAAG	
ncRNA13-F	GTGTCTGACACGGCCCT	Amplification primers of ncRNA13 for qRT-PCR detection
ncRNA13-R	GCACCATTACCGACCGAACG	
ncRNA14-F	AAGCTGGGACGACCC	Amplification primers of ncRNA14 for qRT-PCR detection
ncRNA14-R	GTCGTCCCCTGAATACAA	
ncRNA15-F	GACCGACAGAGAAAAAAGAC	Amplification primers of ncRNA15 for qRT-PCR detection
ncRNA15-R	GCTATCTTTAAAACCATAGC	
ncRNA16-F	GCGGTAACAATTTTGCCTTC	Amplification primers of ncRNA16 for qRT-PCR detection
ncRNA16-R	GTATGGGGCAAAGCCAAGT	
ncRNA17-F	AGGGTGCCAGATAACGTCTG	Amplification primers of ncRNA17 for qRT-PCR detection
ncRNA17-R	TGGAGTTTACCATGCCACAA	
ncRNA18-F	AGTTACTGATACCGAGCACG	Amplification primers of ncRNA18 for qRT-PCR detection
ncRNA18-R	CCCTTGACATAAGCACACGG	
ncRNA19-F	ATGCAGGAACTTTTGCAGCTT	Amplification primers of ncRNA19 for qRT-PCR detection
ncRNA19-R	GGTTTCGGTACTTTCACTGTAC	
ncRNA20-F	GCAACGCTAACTTGTT	Amplification primers of ncRNA20 for qRT-PCR detection
ncRNA20-R	CTTGCCAGCGACATCC	

ncRNA21-F	GAATACGCGCAATTGTT	Amplification primers of ncRNA21 for qRT-PCR detection
ncRNA21-R	AGCCACACTAAGATTTG	
ncRNA22-F	CAAACATTCACATTTGCC	Amplification primers of ncRNA22 for qRT-PCR detection
ncRNA22-R	AATTGGAGGCTATTGTCGTTCT	
ncRNA23-F	TCTGGAACAAGCTGAAAAATTG	Amplification primers of ncRNA23 for qRT-PCR detection
ncRNA23-R	TTTGATGTCAAAACACATTCAAAG	
ncRNA24-F	CTGTGGACAGGGAATCATCA	Amplification primers of ncRNA24 for qRT-PCR detection
ncRNA24-R	TTGACCTTCAGCAAGGGTTT	
ncRNA25-F	CCTTAACGTCTAAGTCATGTGCT	Amplification primers of ncRNA25 for qRT-PCR detection
ncRNA25-R	GTTGCATAGTAACACCTTTCCAA	
ncRNA26-F	TCATCACCATCACCCTGACT	Amplification primers of ncRNA26 for qRT-PCR detection
ncRNA26-R	CCTTCCGGGGTTTTAGTTGT	
ncRNA27-F	TGCTTTTCTTTGATGTCCCCA	Amplification primers of ncRNA27 for qRT-PCR detection
ncRNA27-R	AACCCGACAGTTATTTCACC	
ncRNA28-F	CACCAAGTCATTGGGGA	Amplification primers of ncRNA28 for qRT-PCR detection
ncRNA28-R	ATTACGGCATCTGTACCACG	
ncRNA29-F	GCCTTATGGCTGAGATGAA	Amplification primers of ncRNA29 for qRT-PCR detection
ncRNA29-R	ACCTTGACTTCCCTACGCTG	
ncRNA30-F	CCGAAAAATAAAGAACTAG	Amplification primers of ncRNA30 for qRT-PCR detection
ncRNA30-R	AAAGGCAAATCCAAGGGA	
ncRNA31-F	CAGGTGAATACAACGT	Amplification primers of ncRNA31 for qRT-PCR detection
ncRNA31-R	CATCGGTGTAATAATGG	
ncRNA32-F	GAAGGCACGACATTGCT	Amplification primers of ncRNA32 for qRT-PCR detection
ncRNA32-R	AAAAGCCAGCACCCGAGCT	
ncRNA33-F	TAAGCATTTTGTTGCGCA	Amplification primers of ncRNA33 for qRT-PCR detection
ncRNA33-R	GACGCACCATTTCTCTTTCTTT	
ncRNA34-F	TACTTTAGCGCCTAATTCAAGG	Amplification primers of ncRNA34 for qRT-PCR detection
ncRNA34-R	CTCAGGCGCTACTTTTCGTT	
ncRNA35-F	ATTTATCTGGCTTAGGGAAATGG	Amplification primers of ncRNA35 for qRT-PCR detection
ncRNA35-R	TGAGAAAGTCTGAGTGAGTTCTCG	
ncRNA36-F	ATGCCGTAAGCAACATTTTAC	Amplification primers of ncRNA36 for qRT-PCR detection
ncRNA36-R	AGCCAGATAAATCATTTATCTGGC	
ncRNA37-F	TTCCCGTTTAGCGTTATCCT	Amplification primers of ncRNA37 for qRT-PCR detection
ncRNA37-R	GACATCTTTGCTGCCATCAG	

ncRNA38-F	GTAGATGCCGAGAAGGCAAC	Amplification primers of ncRNA38 for qRT-PCR detection
ncRNA38-R	TAGCTGCTGCTGGACCTTTT	
ncRNA39-F	GGTGCTTGGATCATTTTGGT	Amplification primers of ncRNA39 for qRT-PCR detection
ncRNA39-R	ATTTAGTCATTTCTCTTGACT	
ncRNA40-F	AAAATGGTCTTGTTCAA	Amplification primers of ncRNA40 for qRT-PCR detection
ncRNA40-R	TTCAGCTTAATTCCAGAA	
ncRNA41-F	CCACAATAATGGGTGGGAAC	Amplification primers of ncRNA41 for qRT-PCR detection
ncRNA41-R	TTGAGATATTCCGCTCGTCA	
ncRNA42-F	TTCAACTTTTTATGTCGGTCCA	Amplification primers of ncRNA42 for qRT-PCR detection
ncRNA42-R	AGGTGGTGCCTCACTCCAC	
ncRNA43-F	ATTGACAGGAGCCAGTGCAA	Amplification primers of ncRNA43 for qRT-PCR detection
ncRNA43-R	ATTCATGTGCGATCAATTTCCAT	
ncRNA44-F	CTCTCCCTAGCTTCCGCTCT	Amplification primers of ncRNA44 for qRT-PCR detection
ncRNA44-R	CGCGTCCGAAATTTCTACAT	
ncRNA45-F	TCTCTGAGATGTTTGC	Amplification primers of ncRNA45 for qRT-PCR detection
ncRNA45-R	ACGAATCGGGTATGCTCACA	
ncRNA46-F	TCCTTATCTGTTATCTG	Amplification primers of ncRNA46 for qRT-PCR detection
ncRNA46-R	AACAGCAGGGCCTATCTTCC	
ncRNA47-F	AACGCAATTTTACTATCAAGCCT	Amplification primers of ncRNA47 for qRT-PCR detection
ncRNA47-R	GCGCGGGTTTTCTCTCTAAG	
ncRNA48-F	AGGCGAGGCTCCTATACAAA	Amplification primers of ncRNA48 for qRT-PCR detection
ncRNA48-R	ACACGTCTCGGTTTTAGCACT	
ncRNA49-F	GGTGCTTGGATCAATCTGGT	Amplification primers of ncRNA49 for qRT-PCR detection
ncRNA49-R	TATTTGTTCATTTTCTTGACAGCTC	
ncRNA50-F	GGGTGCTTGGATCAATCTG	Amplification primers of ncRNA50 for qRT-PCR detection
ncRNA50-R	TTGTTCATTTTCTTGACAGCTC	
ncRNA51-F	CAATTGGTTTTATCTGA	Amplification primers of ncRNA51 for qRT-PCR detection
ncRNA51-R	TCTTCGTGTCGCATCG	
ncRNA52-F	AGCGGAGTGATGACTCTC	Amplification primers of ncRNA52 for qRT-PCR detection
ncRNA52-R	AAGCCGATATTTCTATCGG	
ncRNA53-F	TCTGGAACAAGCTGAAAAATTG	Amplification primers of ncRNA53 for qRT-PCR detection
ncRNA53-R	TTTGATGTCAAAACACATTCAAAG	
ncRNA54-F	TTAATTACACACACAAATCGGCA	Amplification primers of ncRNA54 for qRT-PCR detection
ncRNA54-R	TCGTCCACAAATCCCATACG	

ncRNA55-F	TAAGCCGGAACGAAAAGTTG	Amplification primers of ncRNA55 for qRT-PCR detection
ncRNA55-R	TTGGCGGTGCACTATAAAATC	
ncRNA56-F	GTCTCAACGGGGTGCCTTTTT	Amplification primers of ncRNA56 for qRT-PCR detection
ncRNA56-R	CCTGACTCAAATCCCTACGC	
ncRNA57-F	TAATCTCAGGGCGGGGTGAA	Amplification primers of ncRNA57 for qRT-PCR detection
ncRNA57-R	CTGGATCTGCTGACCTTTGC	
ncRNA58-F	TTGAGCCGTATGCGGGGAAA	Amplification primers of ncRNA58 for qRT-PCR detection
ncRNA58-R	TAGCAGCAGCGCATTA	
ncRNA59-F	AGCAATGGTGAGGTGTGAGA	Amplification primers of ncRNA59 for qRT-PCR detection
ncRNA59-R	CCTCTCATTCACCTACTACTGG	
ncRNA60-F	CTTTAACAATCTGGAACAAGCTG	Amplification primers of ncRNA60 for qRT-PCR detection
ncRNA60-R	AGAGTACTTTTCAGATTTGAGATTTTG	
ncRNA61-F	TTTGAGGGTTACGGCCAGTA	Amplification primers of ncRNA61 for qRT-PCR detection
ncRNA61-R	CGATTCAGTAACAGGTGCTCT	
ncRNA62-F	TCTTGTCGGAGTGCCTAGTG	Amplification primers of ncRNA62 for qRT-PCR detection
ncRNA62-R	GGTCGAATCACTCTTGTTCCC	
ncRNA63-F	TTCCCAGAGTGCAGCCTAA	Amplification primers of ncRNA63 for qRT-PCR detection
ncRNA63-R	AGGCTGGTGACCAAAAAATC	
ncRNA64-F	TCTCGTAGGGTACAGAGGTAAGATG	Amplification primers of ncRNA64 for qRT-PCR detection
ncRNA64-R	CCCCAGTCGAAATAAAGTCG	
ncRNA65-F	GAAAGAACGACATTGCTCA	Amplification primers of ncRNA65 for qRT-PCR detection
ncRNA65-R	AAAGCCAGCACCCGGCT	
ncRNA66-F	TTAACAATCTGGAACAA	Amplification primers of ncRNA66 for qRT-PCR detection
ncRNA66-R	CACATTCAAAGTTTGAG	
ncRNA67-F	GTCTGACACTAGACCAA	Amplification primers of ncRNA67 for qRT-PCR detection
ncRNA67-R	ATCGCACCGCGACCAAA	

[a] F, forward primer; R, reverse primer.

[b] Restriction sites are underlined.
