# Supplementary figures and images for: A Novel Non-Coding RNA CsiR Regulates the Ciprofloxacin Resistance in Proteus vulgaris by Interacting with emrB mRNA
Source: Int J Mol Sci. 2021 Sep 30;22(19):10627. doi: 10.3390/ijms221910627 (PMC8508932; doi:10.3390/ijms221910627)

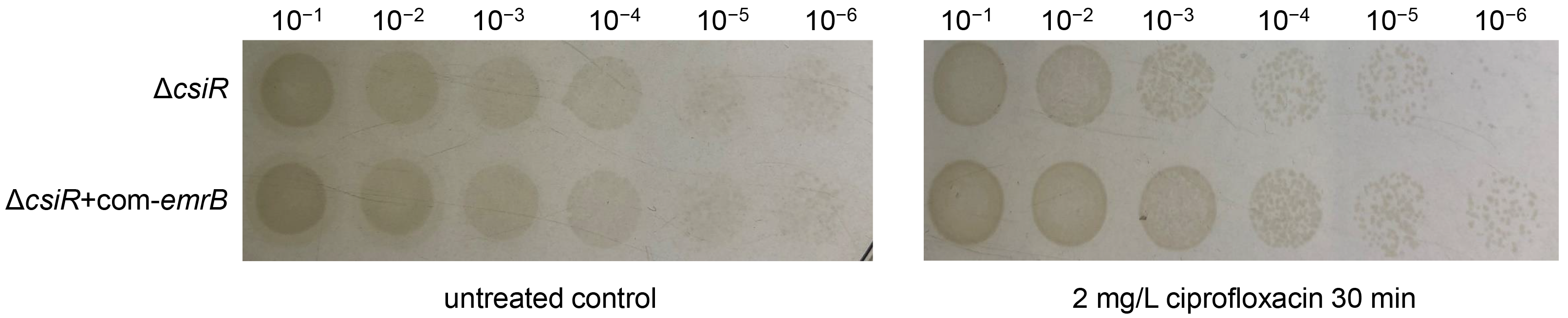

Supplement: Supplementary file 1 [file ijms-22-10627-s001.zip › Figure S1.tif]

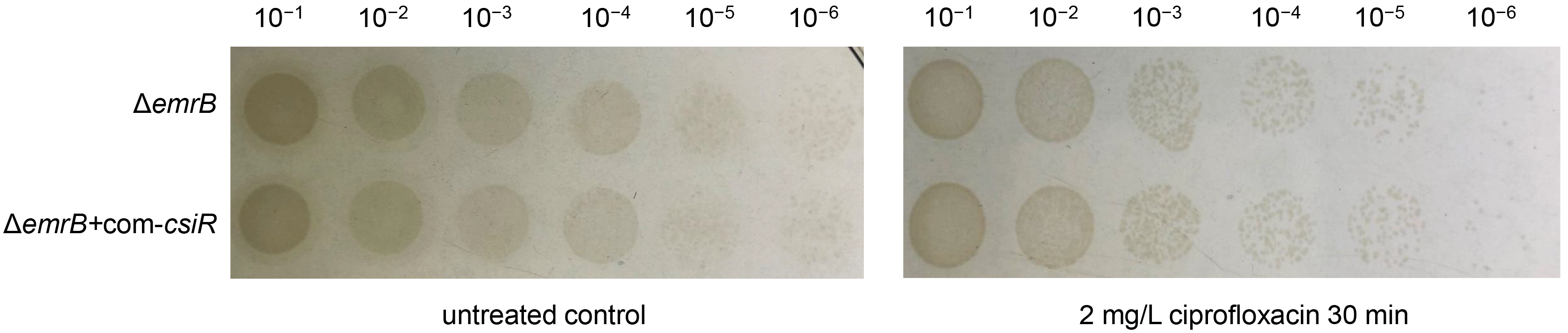

Supplement: Supplementary file 1 [file ijms-22-10627-s001.zip › Figure S2.tif]

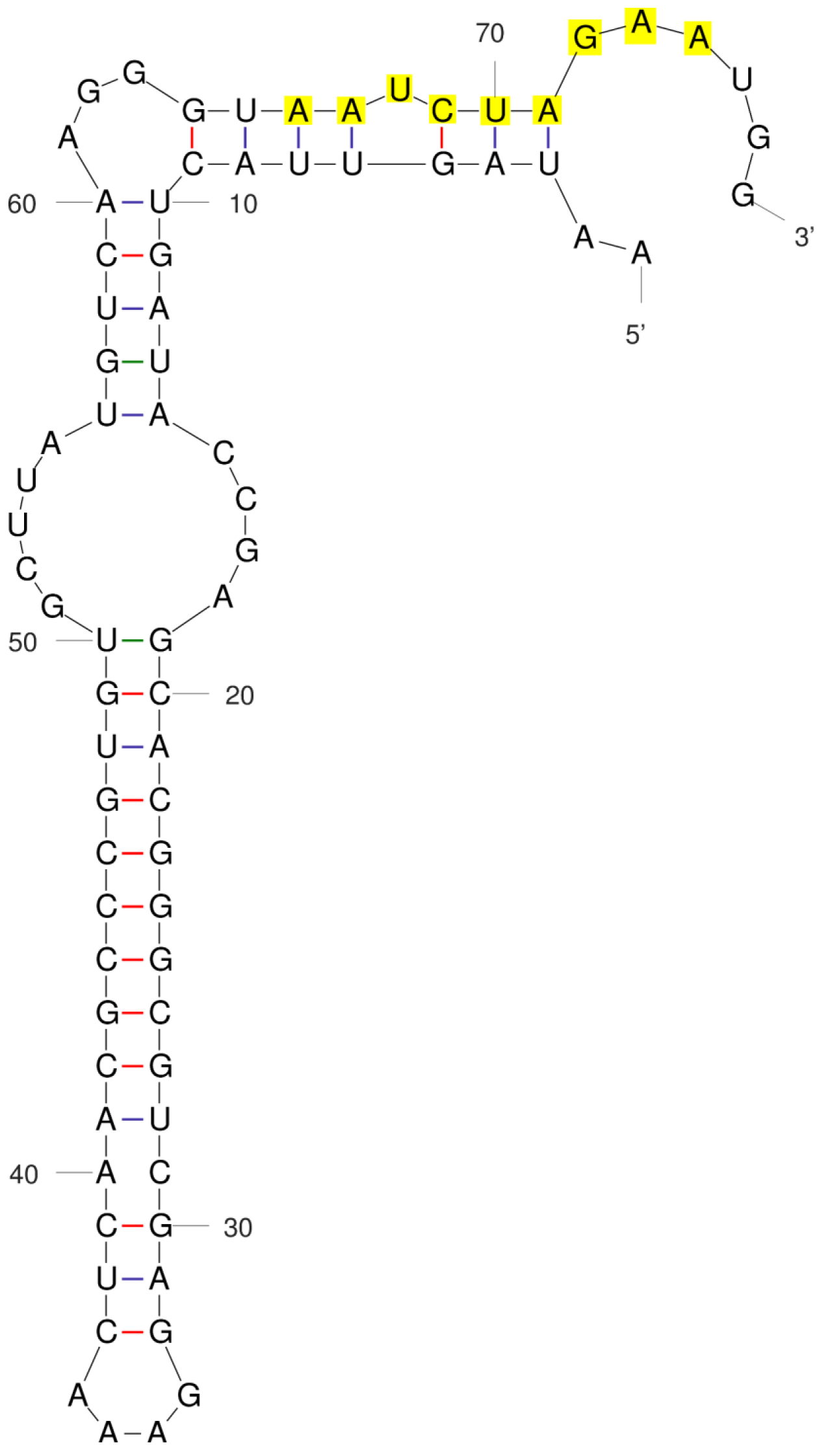

Supplement: Supplementary file 1 [file ijms-22-10627-s001.zip › Figure S3.tif]

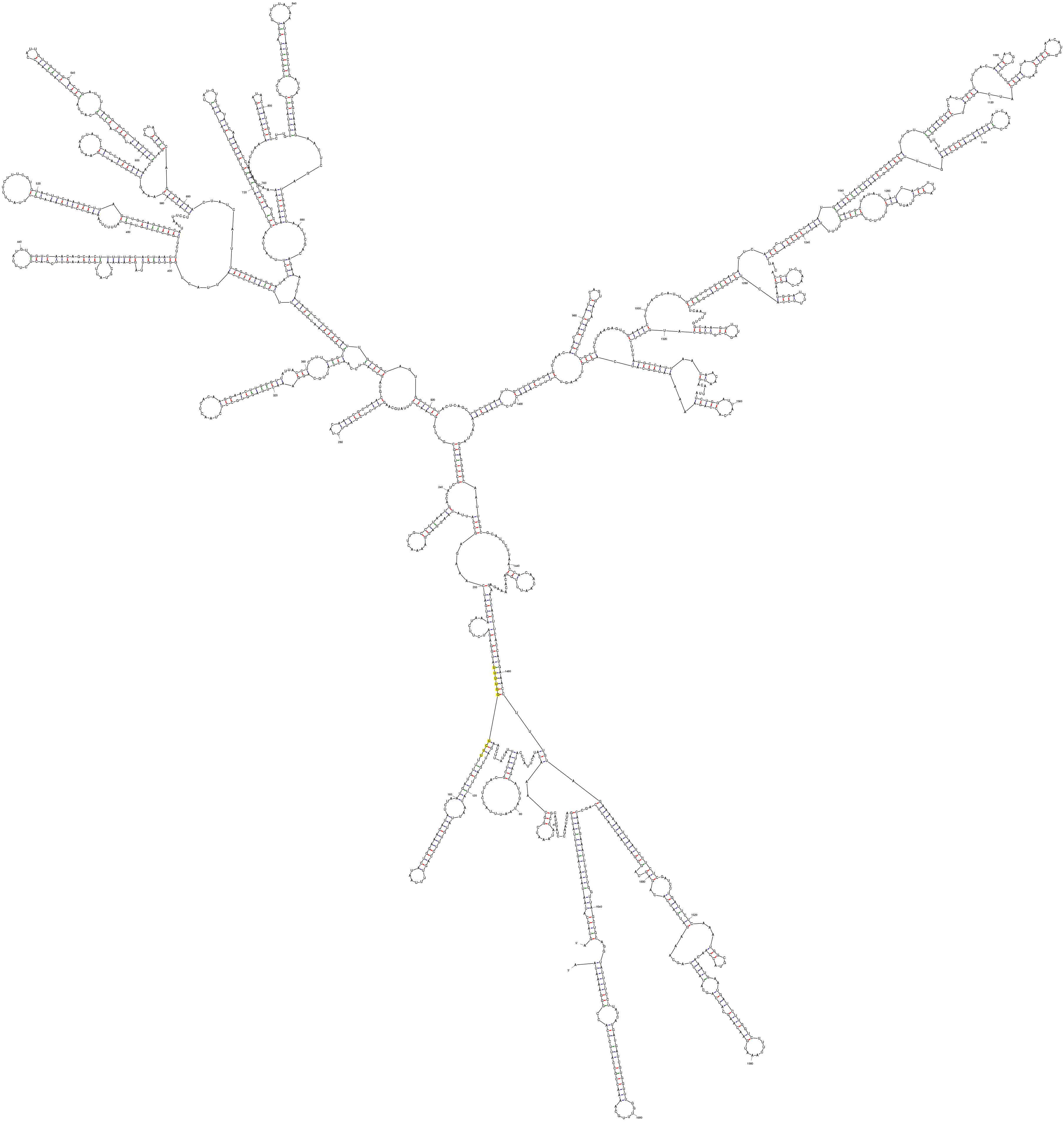

Supplement: Supplementary file 1 [file ijms-22-10627-s001.zip › Figure S4.tif]
